# Association of CLDN molecules with sleep apnea hypopnea syndrome: new biomarker candidates

**DOI:** 10.3389/fneur.2024.1347137

**Published:** 2024-02-21

**Authors:** Dan Liu, Han Meng, Nansheng Wan, Jing Feng

**Affiliations:** Department of Respiratory and Critical Care Medicine, Tianjin Medical University General Hospital, Tianjin, China

**Keywords:** CLDN family, obstructive sleep apnea, biomarker, diagnosis, barrier

## Abstract

**Introduction:**

Obstructive sleep apnea (OSA) is a common sleep-related breathing disorder, and has become a serious threat to public health. Intermittent hypoxia caused by OSA results in a low-grade inflammatory response that leads to impaired mucosal barrier function. Claudin (CLDN) molecules are important for the permeability of the mucosal epithelium. This study aimed to explore whether CLDN molecules can be a potential biomarker of OSA.

**Methods:**

A total of 37 healthy controls and 40 OSA patients underwent a physical assessment for OSA and filled out the STOP-Bang Questionnaire (SBQ) and Epworth Sleepiness Scale (ESS). Clinical specimens of plasma and urine were obtained to observe the difference between OSA patients and healthy controls and diagnostic accuracy of CLDN molecules for OSA.

**Results:**

CLDN1, CLDN2, and CLDN3 molecules in plasma and urine decreased in OSA patients (both *p* < 0.05). The areas under the receiver operating characteristic curve (AUCs) of urinary CLDN1, plasma CLDN1, urinary CLDN2, plasma CLDN2, urinary CLDN3, and plasma CLDN3 were 0.887, 0.724, 0.779, 0.676, 0.828, and 0.665, respectively. The AUC of urinary CLDN1 + CLDN2 + CLDN3 was 0.906 (95% confidence interval (CI), 0.831–0.981). The AUC of plasma CLDN1 + CLDN2 + CLDN3 was 0.776 (95% CI, 0.645–0.878). The AUC of urinary CLDN3 + SBQ was 0.899 (95% CI, 0.832–0.967). The AUC of urinary CLDN3 + ESS was 0.896 (95% CI, 0.826–0.966). In addition, Urinary CLDN-3 was negative associated with the severity of OSA.

**Conclusion:**

CLDN molecules are promising as useful biomarkers for OSA, which may be related to the impaired barrier function related to OSA.

## 1 Introduction

Obstructive sleep apnea (OSA) is characterized by repeated closure of the upper airway during sleep, which leads to intermittent hypoxia (IH), hypercapnia, and increased sympathetic nerve activity (1). Currently, the STOP-Bang Questionnaire (SBQ) and other questionnaires are used in many medical centers for initial screening of patients with suspected OSA, followed by polysomnography (PSG) for high-risk patients (2). PSG, which is the gold standard for diagnosing OSA, requires the patient to wear the device all night in a specific room, and the staff must observe the machine at all times, which consumes considerable time and human resources. In addition, the examination is expensive. It is estimated that approximately one in seven of the world's adult population has OSA. However, the diagnosis rate is very low (3), and testing may be one of the many obstacles to diagnosis. Laborious and expensive PSG is a prohibitive treatment for many patients with suspected OSA. Therefore, qualified biomarkers can help streamline the screening and diagnostic processes for OSA and reduce the financial burden of OSA as a serious public health problem.

Studies have shown that the disruption of mucosal barrier is a common pathological process in OSA. There is evidence to suggest that IH cause changes in the blood-brain barrier (BBB) through oxidative stress, oxygen sensors, increased inflammation, and also influences microvessel permeability of BBB (4). Baronio et al. (5) report higher overall brain water and lower levels of aquaporin 1 in the hippocampus and cerebellum of mice exposed to chronic IH. The pathological damage of intestinal mucosa has also been confirmed. In the presence of hypoxia, intestinal dysfunction leads to necrosis and detachment of the intestinal mucosal epithelium. Once the intestinal mucosa is damaged, the permeability of the intestinal barrier changes. Hypoxia can induce inflammation, and tissues with inflammatory reactions often exacerbate hypoxia, which is a positive feedback phenomenon (6). At the same time, the inflammatory response caused by OSA can also damage the lung mucosa and vascular intima, leading to more serious comorbidities (7).

The CLDN protein family is an important junction protein in barrier function. Currently, more than 20 subtypes of CLDN proteins related to mammals have been identified (8). Partial subtyping of genes has identified defects that are associated with diseases (9), and differential expression of eight subtypes has been found in various diseases (10). However, there is currently not a lot of research on CLDN molecules and OSA. Given the importance of disruption of mucosal barrier in OSA, more studies focus on OSA and barrier function are warranted. Therefore, the aim of our study was to explore the changes of CLDN molecules in patients with OSA, and whether the CLDN molecules can be a biomarker for assessment of OSA.

## 2 Materials and methods

### 2.1 Study design and participants

The present investigation was a study that enrolled consecutive adults who underwent an in-laboratory sleep recording and were diagnosed with OSA from July 2022 to December 2022 at the Sleep Medical Center, Tianjin Medical University General Hospital (Heping, China). All data were anonymous and complied with the requirements of authorities for personal data protection. The study protocol was approved by the Ethical Committee of Tianjin Medical University General Hospital (IRB2019-WZ-175). Healthy participants were also recruited. They underwent PSG to assess whether they should be classified as healthy controls or OSA patients. No participant was undergoing continuous positive airway pressure treatment or had any other lung diseases.

### 2.2 Sleep monitoring

All participants underwent a full night of sleep recording at the Sleep Medical Center. They were allowed to follow their habitual sleep time from 21:00 to 22:00 to 06:00–07:00 hours. Each patient's sleep was continuously monitored using a PSG device (Alice 5; Philips Respironics, Murrysville, PA, USA) by two technicians. Sleep parameters were scored manually using the American Academy of Sleep Medicine Manual v2.3 2016. Respiratory sleep patterns were studied according to the recommendations of the American Academy of Sleep Medicine. The PSG parameters evaluated included sleep latency, sleep efficiency, total sleep time, and duration of each sleep stage. Apnea was defined as the cessation of airflow for at least 10 s in the presence of respiratory effort. Hypopnea was identified as a >30% reduction in airflow for at least 10 s and was associated with either a >3% decrease in oxygen saturation or arousal. The apnea–hypopnea index (AHI) was calculated as the average number of apnea and hypopnea events per hour. Participants with OSA were diagnosed according to an AHI of >5, whereas those with an AHI of <5 were diagnosed as primary snorers. The percentage of time spent in sleep with an oxygen saturation of <90% was defined as T90.

### 2.3 Clinical data and laboratory tests

During the visit, lifestyle questionnaires, SBQ, ESS, medical tests (i.e., electrocardiogram and plasma pressure measurement), anthropometric measurements (i.e., weight, height, and waist and neck circumference), and biochemical tests (fasting plasma glucose, total cholesterol, high-density lipoprotein cholesterol, low-density lipoprotein cholesterol, and triglycerides) were performed. The venous plasma samples and urine samples were collected early in the morning before breakfast. Enzyme-linked immunosorbent assay kits for human CLDN1, CLDN2, and CLDN3 were purchased from J&L Biological Industrial Co., Ltd. (Shanghai, China), and analyses were performed according to the recommended protocols. Participants were considered to have a metabolic syndrome if they had at least three of the following criteria: increased waist circumference (≥94 cm for men and 80 cm for women), increased triglycerides (≥150 mg/dl), decreased high-density lipoprotein cholesterol (<40 mg/dl for men and <50 mg/dl for women), increased plasma pressure (systolic ≥130 and/or diastolic ≥85 mmHg), and increased fasting glucose (≥100 mg/dl) (11).

### 2.4 Statistical analysis

The results for variables that were normally distributed are presented as the means ± standard deviations. The results for variables that were not normally distributed are summarized as medians and compared using the Mann–Whitney *U* test. Student's *t* test was used to compare the means of two independent variables. Spearman's correlation analysis was used to evaluate the relationship between two variables. Receiver operating characteristic (ROC) curves were generated to estimate the area under the curve (AUC), and optimal cutoffs were estimated via the highest Youden's index. Sensitivity, specificity, positive predictive value (PPV), negative predictive value (NPV) and accuracy were calculated through the crosstabs. Binary logistic analysis was used to analyze the ROC curve of the joint indicator. *p* values < 0.05 were considered statistically significant. Error bars were used to indicate the standard deviation. All statistical analyses were performed using SPSS 20.0 (IBM, New York, NY, USA) and Graphpad Prism v.9.0 (California, USA).

## 3 Results

### 3.1 General clinical characteristics of the OSA and control groups

A total of 40 OSA patients and 37 healthy controls were enrolled in the cohort. The OSA patients and controls were matched by age (*p* = 0.065) and sex (*p* = 0.095). The demographic and polysomnographic characteristics of the groups were presented in Table 1.

**Table 1:** Clinical characteristics of the study population.

	**Controls (*n* = 37)**	**OSA (*n* = 40)**	***p*-value**
Age (years)	44.3 ± 12.81	50.33 ± 13.47	0.065
Male, *n* (%)	54.05%	72.50%	0.095
Hypertension (%)	5.41%	70%	<0.001
Diabetes (%)	0%	7.50%	0.091
MetS (%)	8.11%	62.50%	<0.001
TG (mg/dl)	129.23 ± 61.64	190.35 ± 96.67	0.002
HDL-C (mg/dl)	54.94 ± 16.12	41.37 ± 11.97	<0.001
BMI (kg/m^2^)	27.03 ± 6.71	29.85 ± 5.08	0.043
Neck circumference (cm)	38.03 ± 3.22	41.68 ± 3.49	<0.001
Waist circumference (cm)	93.30 ± 7.75	103.9 ± 11.24	<0.001
SE (%)	87.01 ± 7.98	76.94 ± 11.86	<0.001
AHI (events/h)	2.43 ± 1.35	54.51 ± 27.69	<0.001
Arousal (events/h)	8.52 ± 4.82	30.58 ± 22.50	<0.001
AI (events/h)	0.55 ± 0.71	31.81 ± 31.11	<0.001
ODI (events/h)	2.04 ± 2.01	44.85 ± 26.69	<0.001
Mean SpO_2_ (%)	96.59 ± 1.07	92.08 ± 3.25	<0.001
SPO_2_ min (%)	91.89 ± 2.58	72.75 ± 12.54	<0.001
T90 (%)	0	17.76 ± 19.98	<0.001
Plasma CLDN1 (ng/ml)	371.40 ± 44.34	332.59 ± 47.05	0.0006
Urine CLDN1 (ng/ml)	350.53 ± 39.17	269.58 ± 51.43	<0.001
Plasma CLDN2 (ng/ml)	2.21 ± 0.27	2.20 ± 0.29	0.0084
Urine CLDN2 (ng/ml)	2.10 ± 0.28	1.71 ± 0.33	<0.001
Plasma CLDN3 (ng/ml)	4.98 ± 0.64	4.45 ± 0.66	0.0131
Urine CLDN3 (ng/ml)	4.82 ± 0.63	3.70 ± 0.84	<0.001
SBQ	2.18 ± 1.25	3.33 ± 1.01	0.0034
ESS	9.81 ± 3.67	13.88 ± 3.53	<0.001

Data are shown as mean ± SD or median (IQR).

OSA, obstructive sleep apnea; MetS, metabolic syndrome; TG, triglycerides; BMI, body mass index; HDL-C, high density lipoprotein cholesterol; AHI, apnea-hypopnea index; SpO_2_ min, minimum peripheral capillary oxygen saturation; T90, total sleep time spent with oxygen saturation below 90%; AI, apnea index; ODI, oxygen desaturation index; SE, sleep efficiency; SD, standard deviation; SBQ, OP-Bang Questionnaire; ESS, Epworth Sleepiness Scale.

### 3.2 There were significant differences in plasma and urinary CLDN1, CLDN2, and CLDN3 between the OSA and control groups

**Figure 1:**
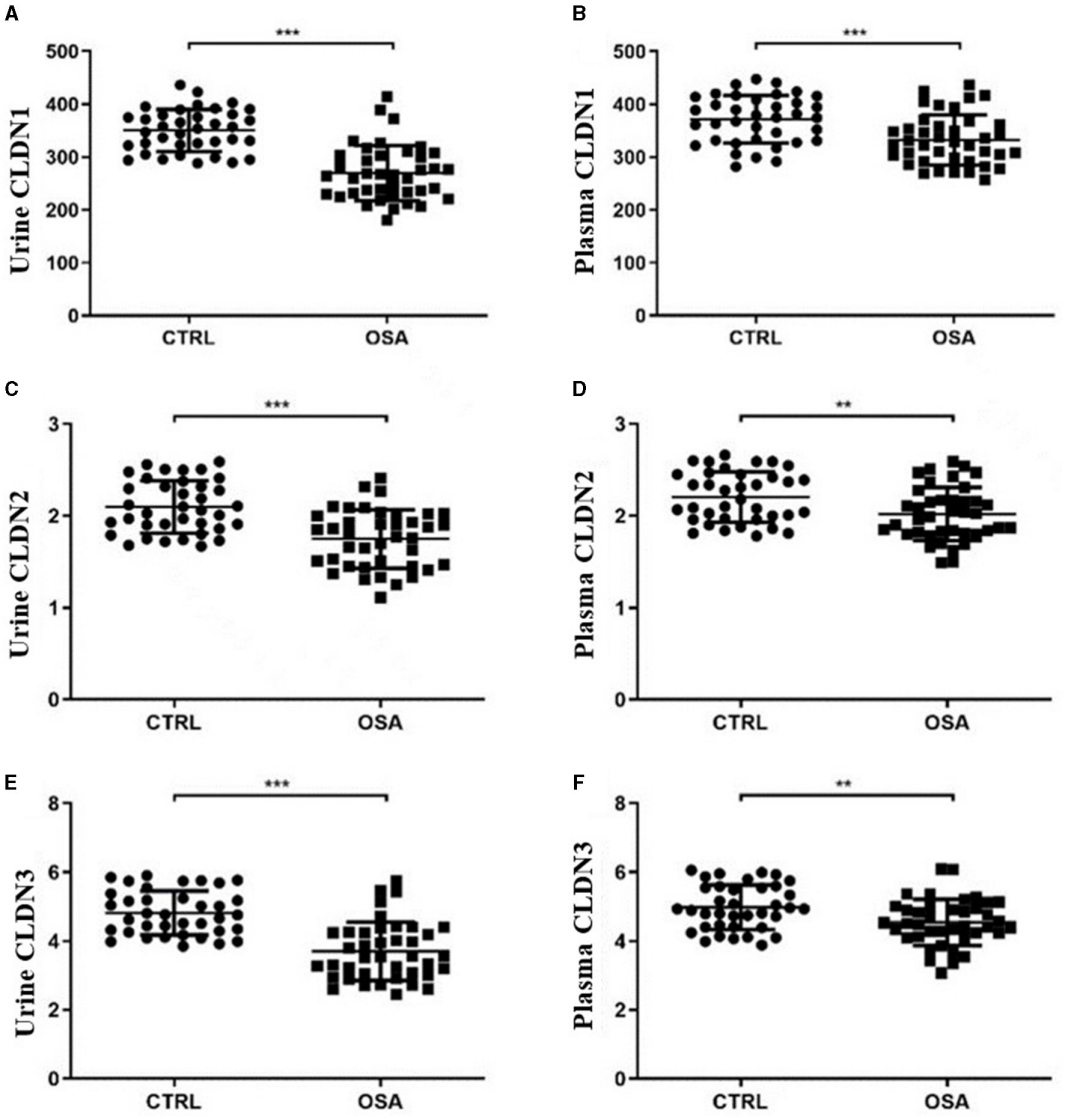
Scatter plot of CLDN molecules in the OSA and control groups. Differences in the expression of CLDN1 in **(A)** urine and **(B)** plasma. Differences in the expression of CLDN2 in **(C)** urine and **(D)** plasma. Differences in the expression of CLDN3 in **(E)** urine and **(F)** plasma. OSA, obstructive sleep apnea. *p < 0.05, **p < 0.01, ***p < 0.001.

The plasma CLDN1, CLDN2, and CLDN3 levels were significantly decreased between the OSA and control groups (*p* = 0.006, *p* = 0.0084, and *p* = 0.0131). The urinary CLDN1, CLDN2, and CLDN3 levels were significantly decreased between the OSA and control groups (both *p* < 0.001; Table 1; Figure 1).

### 3.3 The diagnostic efficacy of CLDN molecules in OSA

We used ROC curves to assess the diagnostic efficacy of CLDN molecules for OSA. The AUC of urinary CLDN1 was 0.827 (95% CI, 0.811–0.964), with a sensitivity of 100%, specificity of 67.5%, PPV of 76.9%, NPV of 100%, and accuracy of 84.4%; the AUC of plasma CLDN1 was 0.724 (95% CI, 0.611–0.837), with a sensitivity of 62.2%, specificity of 75%, PPV of 72.9%, NPV of 64.3%, and accuracy of 68.4%; the AUC of urinary CLDN2 was 0.779 (95% CI, 0.679–0.880), with a sensitivity of 100%, specificity of 42.5%, PPV of 65.3%, NPV of 100%, and accuracy of 72.4%; the AUC of plasma CLDN2 was 0.676 (95% CI, 0.557–0.795), with a sensitivity of 85.6%, specificity of 42.5%, PPV of 61.7%, NPV of 73.3%, and accuracy of 64.9%; the AUC of urinary CLDN3 was 0.828 (95% CI, 0.735–0.922), with a sensitivity of 100%, specificity of 56.1%, PPV of 71.1%, NPV of 100%, and accuracy of 78.9%; the AUC of plasma CLDN3 was 0.665 (95% CI, 0.543–0.786), with a sensitivity of 70.3%, specificity of 57.5% PPV of 64.1%, NPV of 64.2%, and accuracy of 64.1%. It can be seen that the predictive power of urinary CLDN1 is the best among single indicators.

**Figure 2:**
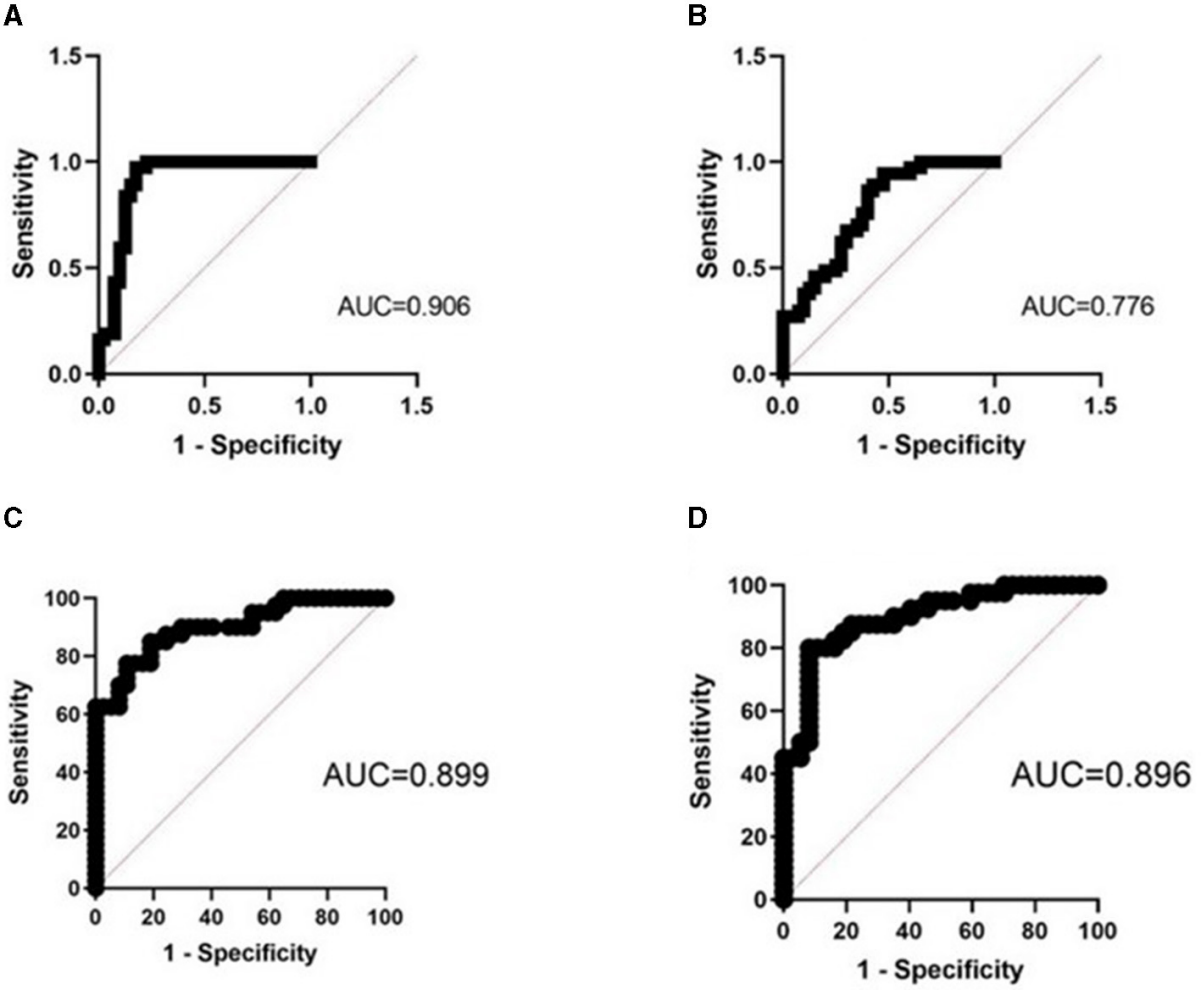
ROC curve of the joint indicator. **(A)** Combined ROC curve of urinary CLDN1 + CLDN2 + CLDN3. **(B)** Combined ROC curve of plasma CLDN1 + CLDN2 + CLDN3. **(C)** Combined ROC curve of urinary CLDN3 + SBQ. **(D)** Combined ROC curve of urinary CLDN3 + ESS. AUC, area under the ROC curve; ROC, receiver operating characteristic; ESS, Epworth Sleepiness Scale; SBQ, STOP-Bang Questionnaire.

The AUC of urinary CLDN1 + CLDN2 + CLDN3 was 0.906 (95% CI, 0.831–0.981), with a sensitivity of 97.3%, specificity of 82.5%, PPV of 85.7%, NPV of 96.6%, and accuracy of 90.2%; the AUC of plasma CLDN1 + CLDN2 + CLDN3 was 0.776 (95% CI, 0.645–0.878), with a sensitivity of 95.5%, specificity of 52.5%, PPV of 68.5%, NPV of 91.5%, and accuracy of 74.8%; the AUC of plasma CLDN3 + urinary CLDN3 was 0.872 (95% CI, 0.786–0.959), with a sensitivity of 95.5%, specificity of 52.5%, PPV of 68.5%, NPV of 91.5%, and accuracy of 74.8%. Among the joint indicators, urinary CLDN1 + CLDN2 + CLDN3 has the best predictive ability. And the predictive ability of the combined index is higher than that of the single index (Table 2; Figure 2).

**Table 2:** Accuracies of indicators in the diagnosis of OSA.

**Markers**	**AUC (95% CI)**	**Cutoff value**	**Sensitivity (%)**	**Specificity (%)**	**PPV (%)**	**NPV (%)**	**Accuracy (%)**
Urinary CLDN1 (ng/ml)	0.887 (0.811–0.964)	258.7	100.0	67.5	76.9	100	84.4
Plasma CLDN1 (ng/ml)	0.724 (0.611–0.837)	359.41	62.2	75	72.9	64.3	68.4
Urinary CLDN2 (ng/ml)	0.779 (0.679–0.880)	1.7	100.0	42.5	65.3	100	72.4
Plasma CLDN2 (ng/ml)	0.676 (0.557–0.795)	1.9	85.6	42.5	61.7	73.3	64.9
Urinary CLDN3 (ng/ml)	0.828 (0.735–0.922)	3.83	100.0	56.1	71.1	100	78.9
Plasma CLDN3 (ng/ml)	0.665 (0.543–0.786)	4.65	70.3	57.5	64.1	64.2	64.1
Urinary CLDN1 + CLDN2 + CLDN3	0.906 (0.831–0.981)	–	97.3	82.5	85.7	96.6	90.2
Plasma CLDN1 + CLDN2 + CLDN3	0.776 (0.645–0.878)	–	95.5	52.5	68.5	91.5	74.8
Plasma CLDN3 + urinary CLDN3	0.872 (0.786–0.959)	–	95.5	52.5	68.5	91.5	74.8

Cutoff values were selected based on Youden's index.

AUC, area under the receiver operating characteristic curve; CI, confidence interval; OSA, obstructive sleep apnea; PPV, positive predictive value; NPV, negative predictive value.

### 3.4 The diagnostic efficacy of CLDN molecules combined SBQ or ESS in OSA

The AUC of SBQ was 0.754 (95% CI, 0.644–0.864), with a sensitivity of 70.7%, specificity of 59.4%, PPV of 65.3%, NPV of 65.2%, and accuracy of 65.3%; the AUC of ESS was 0.786 (95% CI, 0.682–0.891), with a sensitivity of 72.5%, specificity of 73%, PPV of 74.4%, NPV of 71.7%, and accuracy of 72.7%; the AUC of urinary CLDN3 + SBQ was 0.899 (95% CI, 0.832–0.967), with a sensitivity of 85%, specificity of 81.1%, PPV of 82.9%, NPV of 83.3%, and accuracy of 83.1%; the AUC of urinary CLDN3 + ESS was 0.896 (95% CI, 0.826–0.966), with a sensitivity of 80.0%, specificity of 91.9%, PPV of 91.4%, NPV of 81.0%, and accuracy of 85.7%. We found that the combination of urine CLDN3 molecules improved the ability to predict OSA (Table 3; Figure 2).

**Table 3:** Accuracies of indicators in the diagnosis of OSA.

**Markers**	**AUC (95% CI)**	**Cutoff value**	**Sensitivity (%)**	**Specificity (%)**	**PPV (%)**	**NPV (%)**	**Accuracy (%)**
SBQ	0.754 (0.644–0.864)	3.5	70.7	59.4	65.3	65.2	65.3
ESS	0.786 (0.682–0.891)	14.5	72.5	73	74.4	71.7	72.7
Urinary CLDN3 + SBQ	0.899 (0.832–0.967)	–	85.0	81.1	82.9	83.3	83.1
Urinary CLDN3 + ESS	0.896 (0.826–0.966)	–	80.0	91.9	91.4	81.0	85.7

SBQ, STOP-Bang Questionnaire; ESS, Epworth Sleepiness Scale; OSA, obstructive sleep apnea; and AUC, area under the receiver operating characteristic curve; PPV, positive predictive value; NPV, negative predictive value.

### 3.5 Correlation analysis suggests that urinary CLDN3 can predict the severity of OSA

**Figure 3:**
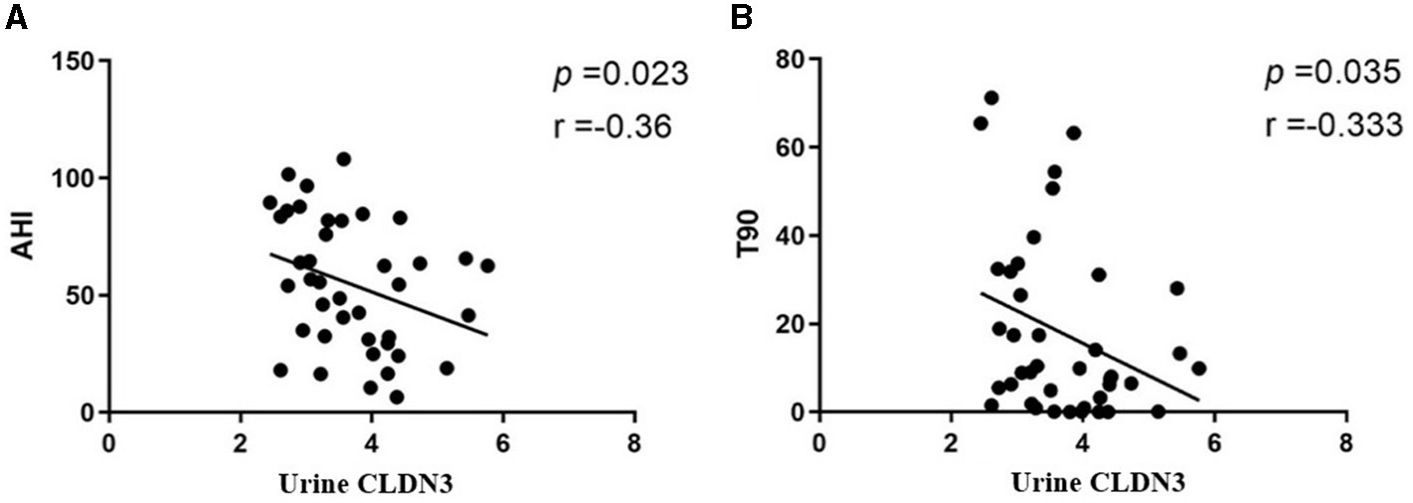
Linear regression plots. **(A)** Urinary CLDN3 and AHI. **(B)** Urinary CLDN3 and T90. AHI, apnea–hypopnea index; T90, total sleep time spent with oxygen saturation of <90%.

We found that urinary CLDN3 was significantly correlated with AHI and T90 (*r* = −0.36, *p* = 0.023; *r* = 0.33, *p* = 0.035; Table 4; Figure 3). This suggested that the more severe OSA, the lower the concentration of urinary CLDN3.

**Table 4:** Correlation analysis of urinary CLDN3, AHI, and T90.

**Factor**		**Urinary CLDN3**	**AHI**	**T90**
Urinary CLDN3	*r*	–	−0.36	−0.33
	*p*	–	0.023	0.035
AHI	*r*	−0.36	–	–
	*p*	0.023	–	–
T90	*r*	−0.33	–	–
	*p*	0.035	–	–

AHI, apnea–hypopnea index; T90, total sleep time spent with oxygen saturation of <90%.

## 4 Discussion

In this study, the association of CLDN1, CLDN2, and CLDN3 molecules with OSA is proposed for the first time. We found that the concentrations of CLDN1, CLDN2, and CLDN3 molecules in the plasma and urine of OSA patients decreased. CLDN1 and CLDN3 are called “pore-sealing CLDNs,” and the increase in the expression of the sealing CLDNs will lead to the increase of the tight junctions of the mucosa. CLND2 is called “pore-forming CLDNs,” which can form paracellular anion/cation pores and water channels, results in reducing epithelial tightness and increasing solute permeability (12). Our study is based on the assumption that IH damages the intestinal mucosal epithelium and thereby impairs expression of CLDN molecules, resulting in decreases in plasma and urine, but the specific mechanism needs to be further explored.

Previous studies have found that IH can cause impairment of barrier function. Baronio et al. reported higher overall brain water and lower levels of aquaporin 1 in the mice exposed to chronic IH (5). A study has shown the brain diffusion alteration in patients with OSA, and neuronal damage and vasogenic edema in the different brain regions of OSA patients due to IH (13). In addition, in the study of intestinal permeability by D-lactate, high intestinal permeability was found in middle-aged male non-obese OSA patients, so there may be subclinical intestinal damage in some OSA patients (14). In this study, we speculated that the decrease of CLDN1 and CLDN2 may lead to the decrease of barrier permeability, while the decrease of CLDN2 may lead to the decrease of water passage, and result in tissue edema. As important molecules in maintaining barrier function, CLDNs have rarely appeared in the study of OSA. Our exploration also provides a promising idea for subsequent basic research.

In addition, the diagnostic efficacy of urinary CLDN3 was 82.8%, and there is a correlation between CLDN3 and the severity of OSA. The AUC of Urinary CLDN1 + CLDN2 + CLDN3 was 90.6%. Urine is an easily obtainable specimen in clinical practice. Therefore, the urine CLDNs are expected to become promising biomarkers for OSA. These findings are very important for further studying biomarkers for the prediction of OSA.

In previous studies, many OSA biomarkers have been discovered, such as I-FABP, D-LA, LPS, and LBP (15). In our previous study, Liu et al. (11) found that neutrophil-to-lymphocyte ratio, lymphocyte, and CD4 counts are associated with “overlap syndrome (OVS)” and have a moderate diagnostic value. These are promising biomarkers for exploring OSA from the perspective of metabolic processes and inflammatory responses. However, there are few researches focus on the barrier function in OSA. We believe that this will help us further explore the impact of OSA on other organ complications.

Combined CLDN molecules, CLDN + SBQ and CLDN + ESS have high AUC, which also provides us with a direction for the screening of OSA. As subjective factors, SBQ and ESS usually have low specificity and accuracy (16). Combined urinary CLDN3 with SBQ and ESS, we found that the specificity and accuracy were significantly increased. In addition, the sensitivity of CLDN1, CLDN2, and CLDN3 molecules in plasma is 100%. Therefore, we believe that adding the CLDN molecules to SBQ and ESS may have more satisfactory results in OSA screening. Future large-scale clinical studies are warranted to confirm our hypothesis.

OSA is frequently associated with comorbidities that include metabolic, cardiovascular, renal, pulmonary, and neuropsychiatric, and there is growing evidence of bidirectional relationships between OSA and comorbidity, especially for heart failure, metabolic syndrome, and stroke (17). And severe OSA was an independent predictor of all-cause death (18). The possible mechanisms include oxidative stress, sympathetic nerve activation, vascular endothelial injury, and endothelial dysfunction. The underlying mechanisms have not yet been explored (19). OSA increases the risk of stroke by 60% (20). Alvarez-Sabin et al. found that in patients with hypertension, moderate-to-severe OSA is independently associated with lacunar silent cerebral infarct (21). Therefore, it is very important to strengthen the screening of OSA in the population. Early detection of patients with moderate and severe OSA can reduce the number of patients with cardiovascular and cerebrovascular diseases and avoid a large amount of waste of public resources.

This study mainly focuses on patients with moderate to severe OSA. We first propose the association of CLDN levels with OSA patients, which provides clues for further understanding of the impaired barrier function related to OSA. Next, we will conduct a large-sample study based on the results of this study to determine whether CLDN molecules can be used to screen patients with moderate and severe OSA, and the potential value of CLDN molecules in mild patients.

## 5 Conclusions

This study mainly explored the association of CLDN1, CLDN2, and CLDN3 molecules with OSA. We found that CLDN molecules have good predictive ability. Urinary CLDN3 was inversely associated with the severity of OSA. The combination of urine CLDN3 and SBQ or ESS can significantly improve specificity for diagnose of OSA, indicating the CLDN molecules as novel biomarker candidates.

## Data availability statement

The original contributions presented in the study are included in the article/supplementary material, further inquiries can be directed to the corresponding authors.

## Ethics statement

The studies involving humans were approved by Ethical Committee of Tianjin Medical University General Hospital. The studies were conducted in accordance with the local legislation and institutional requirements. The participants provided their written informed consent to participate in this study.

## Author contributions

DL: Writing – original draft. HM: Writing – original draft. NW: Writing – review & editing. JF: Writing – review & editing.
